# Protecting Repositories of Indigenous Traditional Ecological Knowledges: A Health-Focused Scoping Review

**DOI:** 10.3390/ijerph22060886

**Published:** 2025-05-31

**Authors:** Danya Carroll, Mélina Maureen Houndolo, Alia Big George, Nicole Redvers

**Affiliations:** 1 Schulich School of Medicine and Dentistry, Western University, London, ON N6G 2M1, Canada; dcarro4@uwo.ca; 2 Faculty of Agricultural Services, Department of Nutrition and Food Sciences, University of Abomey-Calavi, Abomey Calavi 01 BP 4521, Benin; reenmelh@gmail.com; 3 Faculty of Medicine, Dalhousie University, Halifax, NS B3H 4R2, Canada; al525452@dal.ca

**Keywords:** Indigenous peoples, Indigenous knowledge, traditional ecological knowledge, repository, data sovereignty, scoping review, traditional knowledge, climate change

## Abstract

Indigenous Peoples have stewarded Indigenous traditional ecological knowledges (TEK) for millennia. Health-related TEK represents vital knowledge that promotes Indigenous health and wellbeing. Yet, the intergenerational protection of TEK continues to be threatened by various factors, including climate change, which underscores the importance of strengthening and supporting Indigenous-managed TEK repositories. Using a scoping review methodology, we aimed to identify documents for setting up health-related TEK repositories within Indigenous communities. A systematic search was completed in multiple databases—Medline, PubMed, CABI abstracts, Canadian Public Policy Collection, and JSTOR—with manual searches carried out on relevant Indigenous repositories and Google. Content analysis was then carried out with the nine documents meeting our inclusion criteria. We characterized six overarching categories and twelve sub-categories from the included documents. These categories covered impacts on Indigenous TEK repositories resulting from colonial processes, with TEK being seen as diverse, living knowledge protected by longstanding cultural protocols. Concerns surrounding TEK repository management included the need for platforming Indigenous data sovereignty and Indigenous Peoples’ access and ownership. Wise practices of Indigenous-led repository development demonstrated clear examples of data governance processes in action. Indigenous communities were seen to be vital in contributing to key policies and protocols that protect health-related TEK.

## 1. Introduction

Indigenous Peoples have protected and stewarded their traditional knowledge (TK) for millennia. Indigenous traditional knowledges (ITK) are distinctive and contextual, and includes “collective, holistic, community-based, land-informed ways of knowing that are inherently connected with people and the environment” [1,2,3]. Traditional ecological knowledges (TEK) are a form of traditional knowledge that has been acquired over thousands of years by Indigenous Peoples [4,5]. Cajete states that traditional ecological knowledge includes “Indigenous relationships to land, plants, animals, community, self, cosmos, spirit, and the creative animating processes of life” [6]. Traditional ecological knowledges are also inclusive of Indigenous health and wellbeing knowledges (e.g., Indigenous traditional medicine) which are vital to promoting overall good health and balance in all the dimensions of health that many Indigenous Peoples view as being important (i.e., spiritual, emotional, mental, physical).

Globally, TEK are significant to Indigenous health and wellbeing in that it embodies and values Indigenous specific determinants of health [7,8] that encompass Indigenous values, principles, and knowledges that have been instrumental in protecting Mother Earth and Indigenous ways of life. Indigenous scholars often assert that Indigenous Peoples’ relationships to land are rooted in the understanding of TEK, including traditional medicines, Indigenous food systems, and planetary health [7]. Indigenous communities have stewarded their food systems through intergenerational TEK that have sustained and protected traditional foods (e.g., plants, wild game) for millennia [9]. Traditional medicines are embedded in TEK and are integral to the culturally driven health and wellbeing approaches of Indigenous Peoples [10,11]. Indigenous TEK are additionally vital to planetary health, ensuring healthy environments for current and future generations [2]. Planetary health is defined as “a solutions-oriented, transdisciplinary field and social movement focused on analyzing and addressing the impacts of human disruptions to Earth’s natural systems on human health and all life on Earth [12]”. Indigenous TEK is vital to planetary health as it provides approaches and opportunities for Indigenous voices to contribute to key areas including land stewardship, conservation, and research [13]. Indigenous TEK therefore embodies overarching knowledges that include but are not limited to ceremony, land, plants, and food.

Through culturally rigorous intergenerational processes, TEK continues to be passed down within Indigenous communities [14]. Although TEK have most often been passed on orally within Indigenous communities, recently some communities have opted to engage in formal processes and storage of their TEK-related archives and records in various kinds of physical repositories (e.g., audio, video, etc.). Yet, despite some Indigenous communities increasingly using varied repositories for the housing of their TEK, they continue to face many challenges and factors in preserving and protecting their TEK.

In many global contexts, Indigenous Peoples’ knowledge systems have been targeted by colonial policies and practices with the explicit or implicit intent to destroy (i.e., epistemicide) and exploit them [15]. The exploitation of TEK by settler-colonial institutions and governments has been harmful to many Indigenous communities [16]. Harm has included the acquisition of TEK materials often without free, prior, and informed consent (FPIC) from Indigenous Peoples, and often with no consequent benefit back to the communities [17]. Additionally, archival methodology often used by non-Indigenous institutions is based on a paternalistic system that has perpetuated harm to Indigenous communities through limiting access to their own traditional knowledge materials [18]. Furthermore, numerous non-Indigenous institutions including museums continue to house culturally significant and sacred archival materials (e.g., recordings, photographs, videos) belonging to Indigenous communities. When Indigenous Peoples try to repatriate such items, they often face significant barriers, or outright refusal to return sacred items [17].

Conflicts between Indigenous and Euro-Western health beliefs are critical to acknowledge to ensure the long-term protection of TEK which operates under different governance protocols that are relevant to data storage. What may be appropriate in a Western-based data storage system may go against a cultural protocol required in an Indigenous system [19]. For example, certain knowledge may be meant to only be accessible to women and is not appropriate for men to be privy to. These kinds of conventions around Indigenous knowledges are particularly important in the Indigenous context. Given this, there are unique challenges and considerations to developing and strengthening culturally informed data infrastructure and systems within Indigenous communities. Examples of ongoing harms highlight the need for more community-based repositories that are managed by Indigenous Peoples themselves.

Indigenous Nations and communities themselves now generate massive amounts of TEK-related records that may not all be in a central location [20]. Roy and Alonzo state that vital functions of Indigenous archives and repositories include preserving documents, keeping languages and oral traditions alive through recording local Elders, maintaining government records, and preserving vital documents around significant events such as treaty negotiations [21]. Yet, the capacity of an Indigenous community to further organize, develop, and store their TEK-related data and archives in a repository may be limited and presents Indigenous data sovereignty challenges. Additionally, climate change poses further risks to TEK-related repository work within Indigenous communities. For example, the Dene Peoples in northern Canada have had a dense traditional knowledge repository for decades held within the Yamozha K’ue Society (YKS) building located in the K’atl’odeeche First Nation. The knowledge held orally by Dene Elders had been carefully stewarded by the community into varied formats (e.g., tapes, written materials, videos) to ensure proper use and protection. In the early summer of 2023, the YKS building burnt down in a wildfire [22], with a majority of the housed repository materials being destroyed. There were no formalized catalog records of the material or back-ups available, with a majority of the traditional knowledge-related materials completely lost.

Although all TEK-related repositories are important to steward in the face of greater environmental risks to Indigenous communities (e.g., climate-related floods and wildfires), some forms of TEK have unique considerations for data protection and stewardship within repositories. For example, special attention is needed for health and wellbeing-related TEK stewardship including Indigenous traditional medicine knowledges. Health and wellbeing-related TEK are often considered sacred knowledge with strict traditional protocols (i.e., guidelines, procedures) in how this knowledge should be stewarded and passed on. These traditional protocols serve as a mechanism within Indigenous communities to ensure the protection of the medicines as well as the people. In the modern world, Indigenous health and wellbeing knowledge is at risk of both cultural appropriation as well as theft resulting in commercial development (e.g., pharmaceutical patents) without the consent, involvement, or benefit of Indigenous communities [23]. Therefore, important considerations regarding repository management and development for health and wellbeing-related TEK must be considered. With this, despite the importance of protecting and preserving health and wellbeing-related TEK within communities, there is a lack of available Indigenous-led accessible resources on how communities could do this.

### 1.1. Review Objectives

We therefore carried out a scoping review to (1) identify available guidance documents for setting up traditional ecological knowledge repositories within Indigenous communities with a special focus on health and wellbeing-related TEK; (2) identify existing guidance documentation on developing electronic repositories from health and wellbeing-related TEK stored on outdated forms of technology common in Indigenous community repositories (e.g., tapes); (3) reflect on any notable gaps identified in the available guidance documentation on developing Indigenous-led and community-based Indigenous health and wellbeing-related TEK storage repositories.

We specifically chose to use a scoping review methodology to gain a wide scope and understanding of the currently available documents in this area, including identifying key gaps. The scoping review’s broad scope process also supported the identification of some processes which could be used as a starting point for Indigenous communities and researchers working in this area [24]. The Preferred Reporting Items for Systematic Reviews and Meta-Analyses Extension for Scoping Reviews (PRISMA-ScR) provided a standard checklist that guided our reporting [25].

### 1.2. Positionality

It is expected with Indigenous-related research for the authors to position themselves as a form of relational accountability [26,27]. The first author (D.C.) is an Indigenous scholar from the Diné and White Mountain Apache Tribal Nations in the United States. The second author (M.M.H.) is a non-Indigenous African nutrition student. The third author (A.B.G.) is an Indigenous medical student from the Anishinaabeg of Naongashiing First Nation. The last author (N.R.) is an Indigenous and planetary health scholar from the Deninu K’ue First Nation in northern Canada.

## 2. Materials and Methods

### 2.1. Overall Design

The scoping review methodology outlined by Arksey and O’Malley [28] and advanced by Thiessen et al. [29] was used for this scoping review. The PRISMA-ScR reporting guidelines were followed for this review [24]. The protocol for this review was registered on the Open Science Framework (OSF) [30].

### 2.2. Search Terms, Procedures, and Eligibility Criteria

A systematic search strategy was co-developed with a Western University-based librarian (see Table 1) based on what our research questions were. The initial search was conducted up to 13 February 2024 with an updated search carried out up to 7 January 2025. Electronic databases were searched for relevant documents including the following: Medline, PubMed, CABI abstracts, Canadian Public Policy Collection, and JSTOR. Manual searches were also carried out in the Journal of Indigenous Wellbeing: Te Mauri-Pimatisiwin, International Journal of Indigenous Health, and International Indigenous Policy Journal, as well as within the iPortal Indigenous Studies Portal. Google and Google Scholar were searched by reviewing the first 100 articles based on the inclusion criteria. Reference lists of key documents were also reviewed to identify any additional documents of interest relevant to our review.

Documents identified through the search strategy were exported into Covidence review software [31] to facilitate the document selection process. For the purposes of this review, “documentation” refers to any documents from the academic and/or grey literature including organizational documents, toolkits, peer-reviewed articles, and conference proceedings, as well as guidance documents (i.e., guides from various authors including organizations such as the Association of Canadian Archivists). Additionally, “repository” refers to a collection of varied stored materials including but not limited to audio cassette tapes, videos, CDs, and flash drives. Due to varying regional definitions and interpretations of health and wellbeing-related knowledges within Indigenous communities [32,33], health and wellbeing-related TEK is interpreted broadly as any TEK that relates specifically to health and wellbeing knowledges or practices as defined by the relevant Indigenous community within the searched document.

We only included English-language documents due to a lack of resources for translation, and included only those that were available electronically. In addition, we did not limit the type of documents that were included in this review and included toolkits, peer-reviewed articles, and electronic book chapters where accessible, as well as organizational and government documents. Documents were only included if an Indigenous community or Indigenous Nation was included within them. Discussion of some aspect of Indigenous health and wellbeing-related TEK (inclusive of Indigenous traditional medicine) was also required for documents to be included. The documents did not need to be solely focused on health and wellbeing-related TEK but needed to have this included as part of the document. No restrictions were set on publication dates to ensure a broad scope of included documents. Lastly, documents were included if they addressed the need for and/or the development of a TEK-related repository, as well as clearly indicating a process for securing and/or transferring TEK-related data. See Table 2 for a summary of the inclusion criteria for the review.

### 2.3. Article Screening

Covidence software [31] was also used for screening the documents identified from the search strategy. Documents were identified for inclusion by the reviewers (D.C., M.M.H., A.B.G.) using a two-stage process. First, the titles and abstracts of all documents identified through the search strategy were screened by at least two independent reviewers (D.C., M.M.H., A.B.G.) based on the eligibility criteria (see Table 2). Another reviewer supported the resolution of any discrepancies (N.R.). In the second stage of the selection process, the full texts of the included documents were screened by one reviewer (D.C.) based on the eligibility criteria (see Table 2), with a second reviewer screening 25% of the full-text documents to ensure consistency (M.M.H., A.B.G.). Any discrepancies were resolved by discussion with a third reviewer (N.R.).

### 2.4. Data Characterization, Summary, and Synthesis

We carried out data charting in Excel 365 (version 2503) and included the extraction of the following document information: general document information (e.g., citation), geographic location, the Indigenous group the document is intended to apply to (if specified), the intended audience of the document, type of document (e.g., toolkit, organizational document, etc.), the level of community partnership for developing the document (if specified), repository details, the types of stored data involved (e.g., audio, video, etc.), and the TEK referenced.

One reviewer then carried out content analysis (D.C.) to identify key elements from the included documents using the method described by Hsieh and Shannon [34]. Open coding was carried out within the qualitative software analysis tool NVivo (Release 14.23.4) [35], with a second reviewer (M.M.H.) coding 25% of the documents for audit and consistency purposes. A third reviewer was brought in periodically throughout the coding process for discussion and the refining of codes (N.R.). Where health and wellbeing-related TEK was included in only a portion of an included document, only the health and wellbeing-related portions were used for the analysis. We provide a narrative account of our findings organized through the identified content analysis categories as per the Arksey and O’Malley [28] scoping review methodology below.

## 3. Results

**Figure 1:**
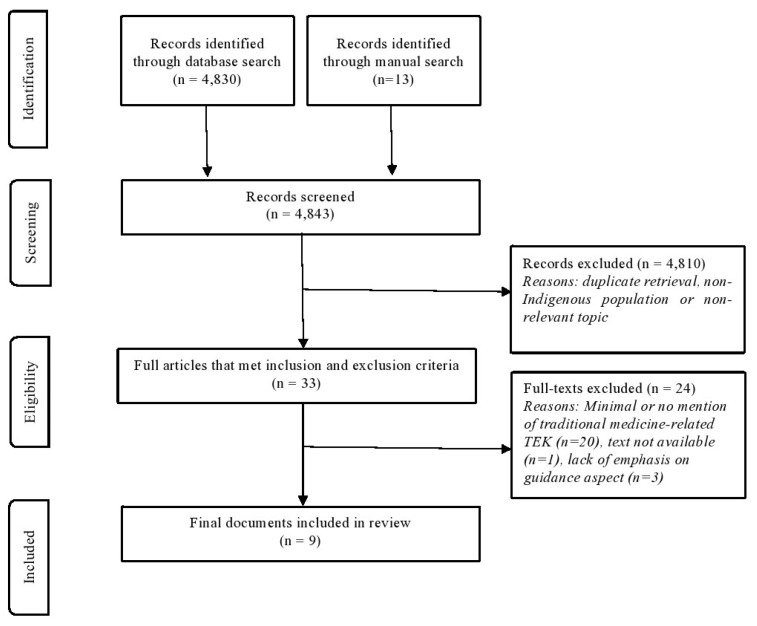
Adapted PRISMA diagram.

A total of 4843 documents were screened for this scoping review, with 9 articles meeting the inclusion criteria for further analysis (see Figure 1). A majority of the final included documents (*n* = 5) were published between 2015 to 2023. The types of documents included in the final analysis were academic journal articles, guidance documents, conference proceedings, and a report. Many of the types of documents identified and included in the final analysis were published from non-Indigenous institutions.

The included documents were published in the United States (*n* = 5), Canada (*n* = 2), Australia (*n* = 1), and the Pacific Islands (Melanesia and Polynesia) (*n* = 1), with a clear gap of documents from other regions around the globe (e.g., Africa, Central and South America). The levels of partnerships outlined in the development of the included documents varied, with a majority of the documents including community-level involvement (*n* = 8). Six of the nine included documents specifically addressed community and academic partnerships. Varied types of Indigenous health and wellbeing-related TEK were addressed in the documents including traditional plants and medicines, land, ceremony, and Indigenous foods. Additional data characteristics for the included documents are listed in Table 3 below, as well as in the Supplementary Materials (see File S1).

Using content analysis, we characterized six overarching categories and twelve sub-categories from the included documents (see Table 4). We review each category and sub-category in further detail below.

### 3.1. Impacts on Indigenous Health and Wellbeing-Related TEK Repositories

Historical experiences and colonial impacts relating to how non-Indigenous-run repositories containing Indigenous items were established are highlighted in this review. Colonial policies were developed that were predicated on the intentional targeting, devaluing, and suppression of Indigenous Peoples’ lands, knowledges, and ways of life [44]. These policies were extremely harmful and destructive to Indigenous knowledges (IK) and the intergenerational processes in which they were passed on. Colonial worldviews greatly influenced the approaches and methodologies that would be implemented in non-Indigenous-run repositories.

*The impacts of Euro-Western-centric worldviews in archives’ development and methodologies* were covered in some documents including how settler governments and institutions pillaged culturally significant objects and materials from Indigenous Peoples. In North America, “[a]nthropologists, ethnographers, linguists, and entrepreneurs collected massive quantities of cultural items and knowledge that were used to document what they believed were the last vestiges of a dying culture…” [19]. Non-Indigenous institutions took tangible and intangible items from Indigenous communities resulting in massive amounts of IK including TEK being held and stored in non-Indigenous institutions to the present day. The First Archivist Circle states that,

[m]ost archives and libraries hold information of a confidential, sensitive, or sacred nature… For Native American communities, the public release of or access to specialized information or knowledge—gathered with and without informed consent—can cause irreparable harm [38].

These nonconsensual actions have been detrimental to Indigenous Peoples on many levels. Powell expresses how this in a sense stole the spirit of many Indigenous Peoples including the Ojibwe [39]. The Association of Canadian Archivists states that “some of the most significant… [Indigenous] records are held by…the federal government, or are in private hands” [37]. Indigenous materials held in non-Indigenous repositories including those relating to historical experiences and significant events have been impacted by colonization.

*Historical trauma and colonization’s effects and impacts on Indigenous health-related TEK repositories* were also discussed within documents. Indigenous archival professionals state that, “[v]irtually every Indigenous society has traditions and laws regarding specialized knowledge, yet these practices are not recognized by Western law” [38]. The vast number of non-Indigenous entities still housing TEK-related materials continues to perpetuate colonial control. In Canada, many of the archives relating to the history and knowledges of First Nations, Inuit, and Métis Peoples are held by settler governments, institutions (i.e., universities), and churches [37]. Similar to North America, archives of Indigenous Peoples in Australia are often housed in settler institutions including at the Australian Institute of Aboriginal and Torres Strait Islander Studies (AIATSIS). Anderson states that even using terminology such as “archive” has perpetuated colonial power and control within repository contexts [36]. The documents included in this review underscored the importance of supporting Indigenous communities to build and strengthen their own TK repositories.

### 3.2. Indigenous Traditional Knowledge Are Diverse, Living Health-Embodying Knowledges

The documents included in this review highlighted how TK cannot be homogenized as they comes from a plethora of unique and different Indigenous communities. For example, in the article by Malsale et al., they described Samoan TK as “tomai tuufasolo” which is ”TK for farming, fishing, and daily livelihoods, with observation of the behaviors of plants, animals, and the atmosphere”…” [41]. Indigenous knowledges are the fundamental core of many Indigenous Peoples’ ways of life and worldviews.

*Longstanding cultural protocols and the transmission of TEK* were discussed in documents as processes encompassing how Indigenous knowledges are shared. Indigenous communities have elaborate data systems to capture their knowledges and continue to recognize their protocols in the transmission of their health-related TEK. From the initial development, the Karuk Peoples centered their repository in their Tribal codes and laws. The management of their traditional knowledge system was stated to have been “passed on to us by the *ikxaréeyav*, or Spirit People, some of whom were transformed into humans, animals, and natural features at an early time of our ancient past” [19]. Indigenous protocols and laws are recognized within many Indigenous run repositories including through such structures as having advisory groups. For example, Powell describes how an advisory board of Ojibwe Elders, Medicine People, artists, and Tribal historians are key to providing oversight of an Ojibwe repository [39].

The documents in this review additionally discussed various aspects of *TEK related to medicine and health*. Johnson-Jennings et al. described their approach to building a digital Food Wisdom Repository to serve Indigenous populations in the United States, that would “resist, recollect, and reclaim Indigenous ways of health, [and] wellness [42]”. They further discussed the importance of the role of data in framing the overall health narratives about Indigenous Peoples. Non-Indigenous projects have often included narratives that may be damaging as they perpetuate deficits-based narratives about Indigenous Peoples instead of being balanced with strengths-based narratives.

In a conference proceeding by Dr. Jane Anderson, she describes how massive amounts of Aboriginal and Torres Islander Peoples’ TEK are being stored at AIATSIS, a non-Indigenous-managed repository, including culturally sensitive materials containing traditional medicine-related TEK. For example, a film recording of a ceremony is stored there, but the cultural protocols associated with who can view the film were not considered until an Indigenous member from the community it was filmed in identified them [36]. The colonial system’s described “copyright owner” that originally recorded the sensitive material has been a considerable barrier posing challenges for AIATSIS and the community from whom the material originates. Attempting to transfer the rightful ownership back to the Indigenous community is complex due to this barrier. Anderson brings up critical fundamental questions on the access, control, ownership, and authorship of Indigenous health-related TEK items being kept in non-Indigenous-owned or -run repositories.

In an article by Yunes et al., they discuss their Rematriation Project which is aimed at building capacity for community digital archiving in Northwest Alaska [43]. This project seeks to protect Inuit knowledges in a region that “faces the devastating effects of rapidly accelerating climate change…” so “…it is critical that communities not only have the resources to preserve their knowledge but retain, recover, and utilize the data collected by academic institutions” [43]. The Rematriation Project illustrates the urgent need to protect TEK, including health and medicine knowledges, in a region where climate change is rampantly changing Indigenous lands and territories.

### 3.3. Indigenous Data Concerns Around Ethics, Ownership, Use, and Governance in the Management of TEK Archives

Indigenous data ownership and management has been undermined by colonial actions for centuries, as illustrated by the documents in this review. Anderson states that “[m]aterial relating to Indigenous [P]eoples lifestyles and cultures exists as published and unpublished material-with a significant amount already in the public domain… with a more recent trend relating to Indigenous knowledge systems” [36]. Increasing public domain access of Indigenous knowledges without Indigenous governance is concerning, and also underscores the importance of Indigenous Peoples movements towards developing their own repositories.

Various elements around *Indigenous data ethics, theft, misuse, and expropriation* were covered in the documents included in this review. Indigenous data protection was noted to continue to be challenging and complex. Indigenous data even beyond TEK-related data can also be challenging for Indigenous communities to access. McMahon et al. stated that, “First Nations often cannot access administrative data and records about their citizens in the possession of third parties such as government agencies” [40]. Indigenous data concerns also include the constant demand for data for research purposes by outside entities. Yunes et al. assert that “[a] vast amount of Indigenous knowledge currently lives behind academic paywalls and are owned and controlled by academic institutions” [43]. They also point out that the “Research Data Lifecycle” undermines Indigenous data sovereignty by supporting researchers’ needs while excluding the needs of Indigenous communities [43]. This data undermining also includes vital aspects of data sovereignty such as ownership, consent, control, access, and storage length [43]. There are also numerous collections in Indigenous communities that are “in jeopardy of loss from housing and building insecurity, deteriorating infrastructure, mold, inadequate storage, and environmental crises” [43].

### 3.4. Platforming Indigenous Peoples’ Access and Rights to Their Data in Repositories

The documents included in this review further highlighted the ongoing concern among Indigenous communities around being able to access their materials that are held in non-Indigenous repositories. Non-Indigenous institutions often retain and control extensive records from the colonial era, effectively making these records the most widely available information about Tribal Peoples to the academic community—and yet, not to their source Indigenous communities [19].

Many non-Indigenous-managed repositories may also inhibit access and/or estrange Indigenous communities from collections that contain their materials and knowledges. Powell states, “[w]e worked together for several years on a project designed to make the SENAD archive more accessible to Cherokee students and the local community. Long discussions with both [E]lders and young people revealed persistent problems…” [39]. This example exemplifies the need to support Indigenous communities to develop and/or strengthen their own repositories.

The narratives and frameworks that have constituted much of the archival field were noted to be based on Western epistemologies. This highlights the importance of Indigenous Peoples reclaiming these spaces and *countering Western data narratives in the governance and management of repositories*. The Mohawk community of Kahnawà:ke in Canada has conducted considerable work in building their digital, as well as their information and communication technologies (ICT). McMahon and colleagues discuss how Kahnawà:ke developed their large digital community data system which covers vast areas including research, education, and health, as well as land and resources [40]. This example highlights how Indigenous Peoples have the capability to be effective and successful stewards of their data, including health-related TEK, when they have the resources (e.g., funding, infrastructure) to do so.

The documents in this review also highlighted that *unbalanced power dynamics between Indigenous Peoples and government/settler institutions affect data repositories*. Anderson asserted that institutions were established based on colonial power which has affected how these institutions portray Indigenous Peoples [36]. For example, “many institutions in Australia hold early photographic material of Indigenous [P]eople[s] in very compromised and denigrating positions” from an Indigenous perspective [36]. Repositories that were developed by colonial entities and that were put in place to administer and manage these materials have been found to be ineffective and at times inappropriate. The Karuk Tribe et al. stated that, “materials in non-Native repositories have been overwhelmingly cataloged by people without the necessary relevant cultural knowledge. This results in highly generalized and often inaccurate metadata as well as insufficient culturally responsive direction relative to collections management” [19]. This has led to the perpetuation of inaccurate translations of important meanings of TEK and underscores the significance of increasing access and ownership of these repositories for Indigenous Peoples.

### 3.5. Securing and Protecting Indigenous Data in an Honorable Way Is Important

The documents included in this review provided notable guidance to support the protection of health-related TEK in varied Indigenous communities around the globe. Furthermore, documents demonstrated how critical it is to secure and protect Indigenous data. Securing and protecting Indigenous health-related TEK can be seen as requiring a two-fold approach. Some communities have much of their own data (e.g., recordings, photos) that need to be further secured. In contrast, other communities may not have much of their own data housed within their communities but may have culturally significant materials housed in non-Indigenous repositories to which they want greater access, or from which they want formal repatriation of the materials.

*Indigenous data ecosystems and worldviews* have been impacted by many factors including data inequities and exploitation. Indigenous worldviews on materials may differ greatly from a Euro-Western worldview. For example, the Onkwéhonwe Peoples view themselves as “belong[ing] to the ‘property’; it doesn’t belong to [them]” [38]. Repositories that platform relational Indigenous worldviews and voices are vital to securing and protecting Indigenous knowledges as they promote these communities’ ability to exercise data sovereignty [42]. Documents in this review also provided further context on *Indigenous data sovereignty processes, principles, and policies within repositories.* Indigenous communities have their own laws that govern their knowledges and materials. For example, the Karuk Tribe et al. states that, “like other Indigenous [P]eoples, our laws governing material and intellectual property remain unacknowledged, and our oral tradition–based knowledge systems have access restrictions that are not reflected by U.S. and international copyright laws” [19]. Some documents in this review also demonstrated how data governance must be strengthened at the community-level including building capacity within communities to collect their own data. The Rematriation Project discussed by Yunes et al. shows the significance of “capacity building that hones, develops, and complements local skills related to digital archiving and digital literacies…” [43]. They emphasized key aspects of their project goals including digitizing materials from an Iñupiat community to further develop a scalable model for community digital archiving.

### 3.6. Wise Practices and Challenges in Supporting Indigenous-Led Repository Development

The documents included in this review provided some exemplary models of Indigenous-led TEK repository development. These documents also highlighted “wise practices” as opposed to “best practices” which can be problematic as “best practices” are often hierarchical and stem from non-Indigenous worldviews [42]. Wise practices are particularly important in promoting health equity and wellness by centering Indigenous ways of being and knowing [42].

*Challenges in the development and maintenance of data repositories* were also highlighted among included documents. The Karuk Tribe et al. shared challenges in developing and managing their repository including a lack of sustained funding for site maintenance and staffing, as well as the difficulty in confronting ethical questions surrounding cultural responsibilities to protect their knowledge [19]. These challenges highlight the disconnect that may occur when oral-based TEK systems are digitized and stored in non-Indigenous structures. Powell also discussed challenges in the development of an Ojibwe digital repository [39]. He stated that, “[w]e have begun to understand that stories should be more important than categories, that [I]ndigenous systems like the colors associated with the cardinal points in Ojibwe cosmology should be employed instead of sidebars and drop-down menus…” [39]. Anderson further discussed considerations and challenges for enhancing the processes and experiences for Indigenous Peoples that use the AIATSIS repository (75% of their clientele) as they have shifted from being subjects to users [36]. For example, Indigenous Peoples in Australia use the repository to address land issues in the court systems [36].

*Community access to IK repositories and processes for consultation* were covered in documents included in this review. The Sípnuuk repository has shown the significance of the community’s overall obligation “to preserve, perpetuate, and pass on this cultural heritage to…succeeding generations” [19]. This repository has fostered intergenerational relationships between Elders and youth by working with families to digitize their private collections. Consultation with families has fostered a reciprocal process that has engaged families with the repository and enhanced the collections at Sípnuuk. In another example, Powell describes how consultation with Elders and cultural advisors enhanced access to the non-Indigenous repository SENAD which contains many culturally important materials for Cherokee Peoples [39]. The substantial size of the archive itself was originally off-putting to Cherokee Peoples who attempted to use it, so consultation with advisors improved the process [39]. These examples show how culturally driven consultation can contribute to repository development that is responsive to the needs of Indigenous Peoples.

*Decolonizing methodologies and wise practices for Indigenous knowledge repository development* were key components supported by the documents in this review. The Karuk Tribe shared that, “[w]e hope that together, we can inform existing data with culturally informed metadata and begin to tell our own story, the true, never-ending story of the Karuk People” [19]. The *Gi bugadin-a-maa goom* project, as discussed by Powell, is a living museum that “will help to ‘awaken’ the archival material through the art of storytelling” [39]. Powell shared how the project engaged a powerful coalition including Sacred Pipe Carriers, drum keepers, Tribal historians, Ojibwe scholars, digital librarians, curators, and archivists [39]. The project further accentuated the importance of having Indigenous leadership and professionals knowledgeable in repository development.

Indigenous rights to data are discussed by Anderson, as well as the experiences with AIATSIS [36]. Anderson asserted that significant progress has been made regarding cultural material including the development of protocols and ethical issues [36]. For example, the “Galiwin’ku Indigenous Knowledge Centre functions to record and document current cultural practices as well as provide a place for the return of important historical recordings to the community” [36]. Engagement with this community enabled AIATSIS to localize access to materials specific to the remote community. Overall, the documents identified in this review highlighted some of the wise practices associated with community-based TEK repositories.

## 4. Discussion

[Indigenous] communities are encountering more frequent destructive storms, fire, and flooding, putting them and their [T]ribal histories and land stewardship at great risk. It is imperative to create accessible, digital versions of valuable and threatened knowledges [43].

Through this review, we attempted to identify available guidance documents focused on setting up TEK repositories within Indigenous communities with a special focus on health and wellbeing-related TEK. We also aimed to reflect on any notable gaps within the available documentation on developing Indigenous and community-based health and wellbeing-related TEK storage repositories. Varied types of Indigenous health and wellbeing-related TEK were addressed in the documents including traditional plants and medicines, land, ceremony, and Indigenous foods. We characterized six overarching categories and twelve sub-categories from the included documents. These categories covered overarching impacts on Indigenous TEK repositories resulting from colonial processes as well as Indigenous data concerns surrounding TEK repository management, including the need for platforming Indigenous data sovereignty and Indigenous Peoples’ access to their data.

Among the documents included in our review, there was much dialogue on securing and protecting existing health-related TEK. Strengthening Indigenous data sovereignty and governance informed by Indigenous worldviews was noted to be significant to this process. Johnson-Jennings et al. stated that, “Indigenous-led data projects can generate stories that represent an Indigenous worldview, Indigenous knowledges, values, and cultural practice, especially when Indigenous health beliefs conflict with Western beliefs” [42].

Keeping Indigenous data in Indigenous hands was identified in our review as being crucial for community wellbeing. Indigenous health and wellbeing-related TEK are most needed by those communities in which the respective TEK knowledge originates. This ensures the intergenerational transfer of knowledge for both Indigenous and planetary health [2]. This review identified different types of health and wellbeing-related TEK including, as examples, food, plants, and ceremony. The deep interconnectivity between TEK, Indigenous Peoples, and the land was apparent, with health and wellbeing-related TEK being seen to be very broad and encompassing. With this, there is a deep and fundamental link between Indigenous Peoples’ healing, TEK, and the land [45]. “Land-based healing”, for example, is an approach that has been seen to bridge Indigenous Peoples, TEK, and the land to improve Indigenous community health and wellbeing [46,47,48,49]. Food-related TEK is also significant for platforming culturally driven health interventions and overall food sovereignty in Indigenous communities [50]. Many Indigenous Peoples in certain regions of the world still rely heavily on having access to traditional foods, often enabled through their TEK [14]. Indigenous TEK are therefore incredibly imperative to ensure long-term Indigenous health and wellbeing, as well as ensuring health equity.

What was also amplified in our review findings was the understanding that without healthy Indigenous lands and the presence of Indigenous languages, Indigenous knowledge, including health and wellbeing-related TEK, would face significant challenges in both its application and its sustainability [45]. With this, Indigenous land rights as well as cultural continuity (i.e., the intergenerational transfer of TEK) are paramount to Indigenous health and wellbeing. Cultural continuity itself has been found to be a key indicator of overall Indigenous community health and connectedness [51,52,53] including within urban Indigenous communities [54,55]. Indigenous-managed repositories are crucial to better ensure cultural continuity in a modern world, and they must also be considered alongside the living application of Indigenous knowledges premised on land and cultural rights, as well as overall Indigenous self-determination.

The documents in this review clearly illustrated the need for more support for Indigenous communities to lead their own repository development, including more training for Indigenous community professionals. The Association of Tribal Archives, Libraries, and Museums (ATALM) is a non-profit organization in the United States that provides some support for these endeavors [55]. They provide training opportunities and resources on planning, building, and administering “Native Cultural Facilities”. Additionally, regarding repository development, is the need for ongoing Indigenous data governance. One notable framework in this regard is the First Nations Principles of OCAP^®^ [56] which “have become the de facto ethical standard not only for conducting research using First Nations data, but also for the collection and management of First Nations information in general” in Canada [57]. The *Miiyupimatisiiun* Research Data Archives Project (MRDAP) is a community-university partnership project that applied OCAP^®^ principles to digitize and transfer research data to the Whapmagoostui First Nation located in Quebec [58]. MDDAP showed how critical it is to recognize that operationalizing data sovereignty is specific to each community [58]. OCAP^®^ has served as an exemplary model that has informed similar data frameworks and trainings in other countries such as the CARE (Collective Benefit, Authority to Control, Responsibility, and Ethics) Principles [59].

The CARE Principles further support processes that facilitate Indigenous data governance and collective benefit [59]. Although the CARE Principles are relatively new, their application is crucial for Indigenous Nations and governments to develop and strengthen codes that encompass protection of their health and wellbeing-related TEK [60]. Some Indigenous scholars also assert that the CARE Principles must also be considered for the data protection of Tribal citizens living off reservation lands [60]. Carroll et al. additionally states that, “Indigenous Peoples’ rights and wellbeing should be the focus across data ecosystems and throughout data lifecycles in order to minimize harm, maximize benefits, promote justice, and allow for future use” [59]. Platforming Indigenous community values and ethics is therefore essential to ensure that Indigenous Peoples themselves determine the benefits, harms, and potential future uses of data pertaining to them, including data housed within repositories [49].

Some documents in this review broached policy-related elements in regard to Indigenous data protection. Policy relating to Indigenous data protection and ownership is vast and complex with overlapping policies and laws at varying levels (i.e., local, state, international), as well as significant differences in their level of implementation across varied geopolitical contexts [59]. At the international level, the United Nations Declaration on the Rights of Indigenous Peoples (UNDRIP) recognizes the knowledge and cultural rights of Indigenous Peoples in Article 11. Article 11 states that, “Indigenous [P]eoples have the right to practice and revitalize their cultural traditions, and customs. This includes the right to maintain, protect, and develop the past, present, and future manifestations of their cultures, such as archaeological and historical sites, artefacts, designs, ceremonies, technologies, and visual and performing arts and literature” [61].

Some countries such as Canada have formally recognized UNDRIP through federal legislation (although the operationalization of UNDRIP in Canada remains unclear) [62]. Leveraging policy such as UNDRIP is vital to supporting Indigenous data sovereignty, the repatriation of cultural items, and the right of Indigenous Peoples to their knowledges and cultural practices. “The Nagoya Protocol on Access and Benefit-sharing” is an additional international framework recognized by some countries under the Convention on Biological Diversity [63]. The Nagoya Protocol also stipulates the consideration of Indigenous Peoples’ laws and community protocols relating to their knowledge [64].

Further support from non-Indigenous allies and institutions is needed to ensure that the important TEK stewardship role of Indigenous Peoples is recognized, platformed, and actively supported (including through access to resources). Leveraging and mobilizing policy and processes that ensure the long-term protection of Indigenous TEK—including through support for repository development and maintenance—is significant to the longevity, health, and wellbeing of Indigenous Peoples. Indigenous health-related TEK are also paramount in the overall movement towards rebuilding and strengthening Indigenous Nations. More formalized resources and capacity support for Indigenous community-owned TEK repositories are therefore needed across varied geographies, as there was a noted lack of documents in our review outside of high-income-country settings (e.g., Canada, the US, Australia) which made up eight of the nine included documents.

With this, there are clear gaps in the breadth of available work being published on this topic, and more work is needed to better capture other health and wellbeing-related TEK repository work being conducted around the world. Ultimately, Indigenous-community-relevant toolkits and other resources are needed to better support various regions in how to navigate the complexities and considerations around Indigenous-community-owned and -operated repositories relevant to health and wellbeing-related traditional ecological knowledges.

### Limitations and Strengths

Despite our structured search strategy, we may have missed relevant documents pertaining to health and wellbeing-related TEK and repositories. More specifically, we noted that there was a paucity of documents that came directly from Indigenous communities themselves, including Indigenous-led and managed libraries and archives. Therefore, we acknowledge that there may be the potential for guiding documents that Indigenous communities, governments, and organizations have created to address this area that are missing in this review. Given the potential sensitivities around health and wellbeing-related TEK, in particular around traditional medicine, Indigenous communities may have chosen not to make such documents public or available in electronic format. In addition, given the small number of articles identified, with the majority coming from the US and Canada, the information presented should not be seen to be representative of all Indigenous Peoples and their unique regions around the globe. Regardless, this review provides key information from regions that may have greater funding access and capacity (e.g., the US, Canada), compared to other regions (e.g., Africa, Asia), with the identified categories intended to provide some baseline level of information from which more initiatives can grow. We additionally felt it was important to contribute preliminary dialogue on Indigenous-led approaches to protecting health and wellbeing-related TEK given the dearth of synthesis resources currently available on this topic. The authors also recognize that they may bring inherent biases which may have affected the selection of and exclusion of certain documents in this review. We have Indigenous and non-Indigenous authors in this review, however, that come from different contexts, which we feel brings balance and strength to the analysis and interpretation of the review findings. Further research work in this space should be directed at developing more toolkit-based repository resources that can be adapted to regional contexts. Such toolkit-related work should more deeply examine TEK protection in the digital world, including security and cultural knowledge rights, as well as Indigenous data sovereignty and governance related to the topic.

## 5. Conclusions

Our scoping review provided insights into which available guidance documents exist that focus on setting up health and wellbeing-related TEK repositories within Indigenous communities. The available guidance documents addressed the overall protection and security of health and wellbeing-related TEK, the impacts on Indigenous TEK repositories from colonial Euro-Western influences, and ongoing paternalistic processes that have dominated archival spaces and institutions. Indigenous knowledges were highlighted as being diverse, living, health-embodying knowledges that are protected and transmitted through longstanding cultural protocols and laws. Health and wellbeing-related TEK were shown to encompass various elements including those related to Indigenous traditional medicines and traditional foods that are seen to be vital to Indigenous health. Concerns surrounding non-Indigenous repositories that house Indigenous materials were widely discussed and highlighted the importance of ensuring Indigenous Peoples’ access and ownership to their own data and knowledges. Wise practices and models of Indigenous-led repository development demonstrated clear examples of data sovereignty and governance processes in action. Indigenous communities were seen to be vital in contributing to key policies and protocols that protect health and wellbeing-related TEK.

Indigenous communities are supporting key work leading toward the strengthening of data sovereignty and the protection of their knowledges through the policies and frameworks highlighted in this review. There is a continued need, however, to support Indigenous communities in their repository development, including through partnerships with researchers, archivists, etc. It is imperative to transform approaches that have historically been top down by platforming Indigenous voices and culturally driven processes. When Indigenous Peoples can further strengthen and secure their TEK repositories, they are also safeguarding these repositories from growing threats including climate change. Overall, the protection of health and wellbeing-related TEK, including Indigenous traditional medicines, are vital to the health and longevity of Indigenous Peoples and Mother Earth.

## Figures and Tables

**Table 1:** Example Medline search strategy.

Database	Search Terms
Medline	“Indigenous knowledge *” OR “traditional knowledge *” AND digitization OR preserv * OR archiv * OR repositor * AND protocol OR guideline * OR template OR “best practice” AND health OR wellness

**Table 2:** Summary of the inclusion criteria for the scoping review.

	Inclusion Criteria
Source type and date	English language; peer reviewed sources, as well as government and organizational sources (e.g., Indigenous government reports), and toolkits. No limit for publication date.
Traditional ecological knowledges focus	Indigenous health and wellbeing-related TEK as a concept for this review was used as an “umbrella term” for anything specific or related to health and wellbeing-related ecological knowledges (e.g., food, plants).
Population	Documentation is relevant for Indigenous communities with no limits to geographic context.
Process of repository development	Documentation addresses the need for and/or the development of a TEK-related repository, and clearly indicates a process for securing and/or moving TEK-related data.

**Table 3:** Characteristics of the included documents from the scoping review.

Reference	Year	Country	Document Type (* Open Access)	Type of TEK Addressed	Key Findings: Indigenous Repository Development
Anderson [36]	2005	Australia	Conference proceeding	Ceremonial, traditional medicine	Covers key aspects of Indigenous materials held in non-Indigenous repositories including control, access, and ownership. Also provides case studies and digital aspects (i.e., computer use to access repository) to enhance Indigenous access, and issues of custodianship.
Association of Canadian Archivists [37]	2007	Canada	Guidance document	Land, language, food	Introductory guidance on archival concepts, basic requirements of archiving, additional general information on assistance and resources.
First Archivist Circle at Northern Arizona University [38]	2007	United States	Guidance document	Ceremonial, traditional medicine, land	Covers various aspects of archiving Indigenous materials including relationships, intellectual property issues, copyright/repatriation, protocols, reciprocal education, and training. Also provides resources and guidelines for action.
Powell [39]	2007	United States	Academic journal article	Ceremonial, traditional medicine, plants, land	Provides an overview of key aspects of partnerships to build Indigenous digital archives and discusses lessons learned.
McMahon et al. [40]	2015	Canada	Academic journal article	Health, land	Covers various aspects of Indigenous digital data management. Also provides a case study of community data management and data governance strategy.
Karuk Tribe et al. [19]	2017	United States	Academic journal article *	Traditional medicine, food, plants, land	Highlights Indigenous access, control, and repatriation of materials in non-Indigenous repositories. Shares the process of building the Sípnuuk repository including planning, user needs assessment, and establishing protocols and policies for repositories.
Malsale et al. [41]	2018	Pacific Islands	Academic journal article	Climate, land, plants	Provides a case study example of partnerships that platform Indigenous input including protocols for community engagement to collect and store TEK. Also provides an overview of the legal landscape around TEK.
Johnson-Jennings et al. [42]	2019	United States	Academic journal article *	Food, plants, land	Outlines key aspects of Indigenous data sovereignty and provides a theoretical framework for developing the Food Wisdom Repository.
Yunes et al. [43]	2023	United States	Conference proceeding	Land, climate	Provides a case study of building community digital archiving capacity and strategy towards data sovereignty.

**Table 4:** Indigenous traditional ecological knowledge (TEK) repository scoping review categories characterized.

Category	Sub-Category
*Impacts on Indigenous health and wellbeing-related TEK repositories*	-Impacts of Euro-Western-centric worldviews in archive development and methodologies
	-Historical trauma and colonization’s effects and impacts on Indigenous health-related TEK repositories
*Indigenous knowledges are diverse, living, health-embodying knowledges*	-Longstanding cultural protocols and transmission of TEK being a living process
	-TEK related to health and medicine
*Indigenous data concerns around ethics, ownership, use, and governance in the management of TEK archives*	-Data ethics, theft, misuse, and expropriation
*Platforming Indigenous Peoples' access and rights to their data in repositories*	-Countering Western data narratives in the governance and management of repositories
	-Unbalanced power dynamics between Indigenous Peoples and government/settler-colonial institutions affect data repositories
*Securing and protecting Indigenous data in an honorable way is important*	-Indigenous data ecosystems and worldviews
	-Indigenous data sovereignty processes, principles, and policies within repositories
*Wise practices and challenges in supporting Indigenous-led repository development*	-Challenges in the development and maintenance of data repositories
	-Community access to Indigenous knowledge repositories and processes for consultation
	-Decolonizing methodologies and wise practices for Indigenous knowledge repository development

## Data Availability

The original contributions presented in this study are included in the article/Supplementary Materials. Further inquiries can be directed to the corresponding author.

## Supplementary Materials

The following supporting information can be downloaded at: https://www.mdpi.com/article/10.3390/ijerph22060886/s1, File S1: Full data extraction table. The full data extraction file includes information on the Document Reference, Year Published, Document Type, Specific Indigenous group/region, Level of community partnership, Repository name, Type of data addressed, Types of TEK addressed, and a Summary of the document.
